# Relationship between objectively measured conversation time and social behavior in community-dwelling older adults

**DOI:** 10.3389/fneur.2024.1479296

**Published:** 2024-10-07

**Authors:** Noriyuki Kimura, Teruaki Masuda, Takuya Ataka, Etsuro Matsubara

**Affiliations:** Department of Neurology, Faculty of Medicine, Oita University, Oita, Japan

**Keywords:** cohort study, community-dwelling older adults, conversation time, social behavior, wearable sensor

## Abstract

**Background:**

Social isolation is a significant public health concern in aging societies. The association between conversation time and social behavior remains unclear. This study examines whether objective conversation time is associated with social activity frequency in older adults.

**Methods:**

This prospective cohort study enrolled 855 older adults (538 women; mean age, 73.8 years) aged 65 and older, who were followed from 2015 to 2019. All participants wore a wristband sensor to measure conversation time for at least 9 days and an average of 31.3 days per year. Social behaviors were assessed through interviews, and the frequency of engagement in community activities, outings, lessons, or classes and contact frequency were assessed using a self-report questionnaire. The association between conversation time and social behavior was evaluated using multi-linear regression analysis.

**Results:**

Conversation time was significantly associated with the frequency of engagement in community activities and lessons or classes after adjusting for several covariates (*β* = 0.181, 95% confidence interval: 0.107–0.254, *p* < 0.001; β = 0.11, 95% confidence interval: 0.04–0.179, *p* = 0.002).

**Conclusion:**

Objectively measured conversation time using a wearable sensor is associated with social behavior and may be a valuable parameter for social isolation in older adults.

## Introduction

There is growing evidence that social isolation is implicated in an increased risk of poor health, all-cause mortality, depressive symptoms, cardiovascular disease, and cognitive decline (1–3). In recent years, social network sizes and contact with communities, neighborhoods, and families have decreased (4, 5). In particular, older adults frequently experience social isolation due to declining physical mobility, cognitive function, and economic resources or roles in society, as well as the death of their spouses. Globally, more than 25% of adults aged 65 and older experience social isolation (6), making it a significant public health concern in aging societies (7). Social isolation is a major modifiable risk factor for dementia, and social activity can increase cognitive reserve, improve motivation for healthy behaviors, and reduce stress and inflammation (8–10). Therefore, early identification and management of older adults at risk of social isolation is essential for public health. Two distinct terms refer to subjective experience and objective isolation from other social actors: loneliness refers to the perceived experience of being isolated, while social isolation denotes the actual absence of social contact. This study prefers the term “social isolation” over “loneliness.” Social isolation is characterized as a scarcity of social contact and communication with a broader community, neighborhood, and relatives. It is measured by the frequency of social engagement or social contact and social network size, including marital status or family structure, via questionnaires (4, 5, 11). However, the usefulness of subjective self-report questionnaires is limited with respect to consistency and reliability due to recall bias or misclassification among older adults (12, 13). Moreover, the social stigma of social isolation reduces the likelihood of reporting social isolation, resulting in decreased identification of isolated individuals. Developing an objective method to assess social isolation would help address it among older adults. Wristband sensors are cost-effective and non-invasive tools for objectively and continuously measuring lifestyle factors without recall bias in the community (14–17). We have developed a wristband sensor for quantifying conversation time and used it in a prospective cohort study on the association of lifestyle factors with cognitive function in older adults living in the community (16). The random forest regression analysis results show that objective conversation time is non-linearly associated with the global cognitive function. These results lead us to hypothesize that decreased conversation time may be related to cognitive impairment through social isolation in older adults. Several studies have examined the association of objectively measured conversation time using smartphones with loneliness in young adults or patients with schizophrenia spectrum disorders (18–20). To the best of our knowledge, few studies have measured conversation time using wearable sensors in older adults living in the community. Therefore, this study aims to determine whether objective conversation time is cross-sectionally associated with social behavior.

## Methods

### Study participants

This USUKI study is a prospective cohort study designed to explore the risk or protective lifestyle factors for dementia among community-dwelling older adults aged ≥65 years without dementia in Usuki, Oita Prefecture, Japan (16, 17). The criteria for participation included residents of Usuki, good physical and mental health, and independence in activities of daily living. All participants were asked to wear a wristband sensor (Silmee™ W20, TDK Corporation, Tokyo, Japan), except when bathing, for an average of 7–14 consecutive days every 3 months (i.e., four times per year (total study period = 56 days)) considering seasonal differences in lifestyle. We defined valid sensing data as at least 4 days per period and two periods per year, according to a previous study (14). Participants with at least 9 days of measured conversation time per year were included in this study. A total of 855 older individuals (538 women [62.9%]; mean age [standard deviation (SD)]: 73.8 [5.8] years; mean education duration [SD]: 11.8 [2.1] years) met the inclusion criteria and had valid sensing data for research between August 2015 and October 2017. The number of participants with valid conversation data declined during the follow-up period (770 [90.1%] in the second year and 664 [77.7%] in the third year). Moreover, conversation time or social behavior generally remain habitual without change over only a few years. Therefore, we focused on the cross-sectional association of objective conversation time with social behavior in this study. Trained medical staff collected information regarding demographic characteristics, including age, sex, years of education, engagement in community activity, outings, lesson or class frequency, and contact frequency. The valid data on engagement in community activity, outings, lesson or class frequency, and contact frequency were collected from 656, 785, 771, 764 individuals. Mini-Mental State Examination (MMSE) was used to assess the global cognitive function. This prospective study was conducted in accordance with the Declaration of Helsinki and approved by the Ethics Committee of Oita University Hospital (UMIN000017442). Written informed consent was obtained from all participants before participating in this study.

### Wearable sensor data

A novel wristband sensor was developed to measure the duration of a conversation but not its content. The microphone on the sensor continuously captured the sound pressure generated by speech within a 2-m radius from the device at 1-min intervals. The level of sound pressure of the voice at this distance ranged from 55 to 75 dBA. Frequency bands comparable to speech were extracted as sound frames from the surrounding sounds. A conversation is defined as 1 min with four or more speech frames per minute. Although the sound data included speech from nearby people, participation in the conversation was considered necessary for social activity. Therefore, the amount of time spent in conversation was calculated by summing the daily sensing data and averaging them over the total measurement period. We verified the false detection of various sounds, such as noise while clothing, television, working in an office, or commuting, wind, passing vehicles, musical instruments, animals, and appliances. Moreover, the measurement accuracy was evaluated by comparing the sensor data with video observational data on the conversation time in healthy older adults aged 60–80 years. The conversation time measured by the wearable sensor was found to be significantly correlated with that measured via video observation (*r* = 0.8512; *p* < 0.0001, Pearson correlation). Detailed methods and results have been discussed in previous reports (16).

### Questionnaire on social behavior

In this study, social behavior was constructed from the frequency of outings or contact and cognitive or social activity. Data were collected using self-report questionnaires. The frequency of engagement in community activities was measured as a continuous variable. The frequency of outings, lessons or classes, and contact with friends or relatives was measured on a four-or five-point frequency scale as follows: outing (none, 1–2 days a week, 3–4 days a week, and ≥ 5 days a week), lesson or class (the frequency of taking a lesson or class: none, 1–2 days a month, 1–2 days a week, 3–4 days a week, ≥ 5 days a week), and contact with friends or relatives (none, 1–2 days a month, 1–2 days a week, 3–4 days a week, and ≥ 5 days a week). The score of outings is on a 3-point scale from 0 to 3, whereas that of lessons or classes and contact with friends or relatives is on a 4-point scale from 0 to 4.

### Statistical analysis

The association between conversation time and social or cognitive activity was examined as follows. First, the correlation between conversation time and the frequency of engagement in community activities and the score of outings, lessons or class, and contact with friends or relatives was analyzed using Spearman’s rank correlation. Second, a multi-regression analysis was conducted to examine the association between conversation time and the frequency of engagement in community activities and the score of outings, lesson or class frequency, and contact with friends or relatives after adjusting for covariates, including age, sex, and years of education. The IBM SPSS Statistics software package v. 29.0 was used for the statistical analyses. Statistical significance was set at *p* < 0.05.

## Results

### Demographic and clinical characteristics of the participants

Table 1 summarizes the demographic characteristics, conversation times, and social behaviors of the participants. The mean (SD) point of the MMSE score was 28.4 (1.9). The mean (SD) conversation time collection duration with the wristband sensor was 31.3 (7.1) days per year (7.8 days on average every 3 months).

**Table 1:** Clinical and demographic characteristics of all participants.

Characteristic	
Age, years, mean (SD)	73.8 (5.8)
Sex (M: W)	317: 538
Education duration, mean (SD)	11.8 (2.1)
MMSE score, mean (SD)	28.4 (1.9)
Conversation time, min/day, mean (SD)	228.0 (86.6)
Community activity, day/week, mean (SD)	0.9 (1.5)
Number of outings score, mean (SD)	2.41 (0.8)
Lesson or class score, mean (SD)	1.04 (1.5)
Contact with friends or relatives score, mean (SD)	2.2 (1.4)

SD, standard deviation; M, man; W, woman; MMSE, Mini-Mental State Examination.

### Association between conversation time and social behavior

**Figure 1:**
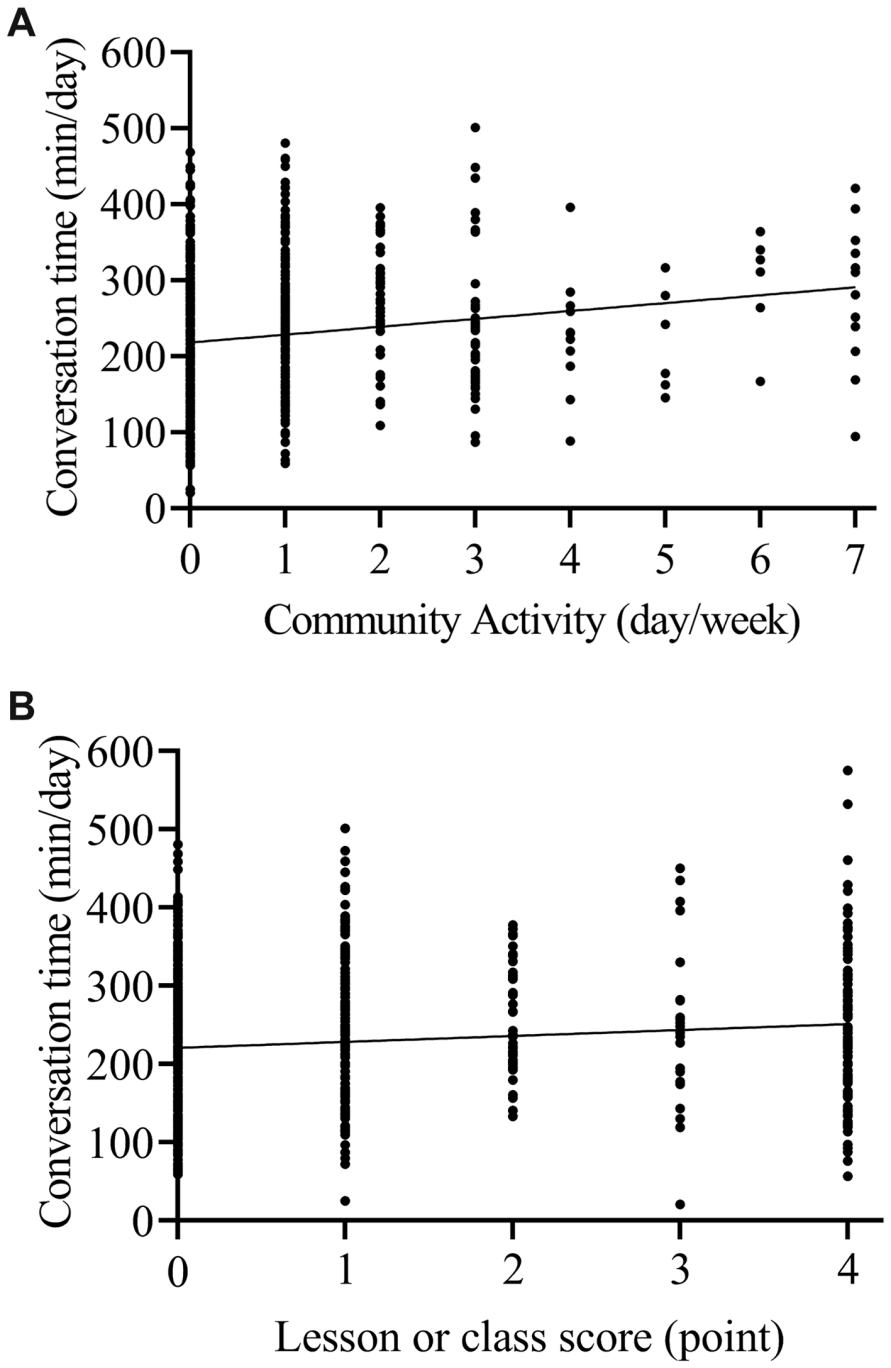
Relationship between objectively measured conversation time and social or cognitive activity. A significant correlation was found between conversation time and the frequency of engagement in community activities **(A)** and lessons or classes **(B)**.

Spearman’s rank correlation results showed that conversation time was positively correlated with the frequency of engagement in community activities and lessons or classes (Table 2; Figure 1; *r* = 0.217, *p* < 0.001 and *r* = 0.168, *p* < 0.001, respectively). Additionally, conversation time remained significantly associated with the engagement in community activities and lessons or classes after adjusting for several covariates in the multi-regression analysis (Table 3; *β* = 0.181, 95% CI: 0.107–0.254, *p* < 0.001 and *β* = 0.11, 95% CI: 0.04–0.179, *p* = 0.002, respectively; *β* = 0.11, 95% CI: 0.04–0.179, *p* = 0.002 and *β* = 0.181, 95% CI: 0.107–0.254, *p* < 0.001, respectively).

**Table 2:** Correlation between conversation time and social or cognitive activity.

	Conversation time	
	*r*	*p*
Community activity	0.217	< 0.001*
Outing	0.018	0.612
Lesson or class	0.168	< 0.001*
Contact with friends or relatives	−0.001	0.968

*p < 0.05 is considered statistically significant.

**Table 3:** Multiple regression model between conversation time and social or cognitive activity.

	Conversation time	
	β (95% CI)	*p*
Community activity	0.181 (0.107, 0.254)	<0.001*
Outing	0.032 (−0.037, 0.1)	0.366
Lesson or class	0.11 (0.04, 0.179)	0.002*
Contact with friends or relatives	−0.015 (−0.084, 0.055)	0.679

*p < 0.05 is considered statistically significant.

## Discussion

We developed a novel wearable sensor that enables the quantification of conversation time. To the best of our knowledge, this is the first study to examine the association between objectively measured conversation time and self-reported social behavior in older adults living in the community. This study provides valuable insight into the relationship between daily living activities and social isolation. Our results show that decreased conversation time is associated with less frequency of engagement in community activities and lessons or classes. We suggest that objective conversation time may be a valuable marker of social isolation. Moreover, the main advantages of this study include the objective measurement of conversations and the large-scale inclusion of community-dwelling older adults.

A key finding of this study was that objective conversation time is positively associated with the frequency of engagement in community activities and lessons or classes. Furthermore, this association remained significant even after controlling for age, sex, and years of education. However, no significant correlation was found between conversation time and the frequency of outings or contact with friends or relatives. These results suggest that conversation time is associated with engagement in community activities or classes rather than simple outings or social contact. Social isolation is defined as a lack of social contact with family and friends or involvement in social, religious, or other outdoor activities beyond social contact. Social isolation is generally assessed by self-report questionnaires regarding social network size, frequency of social contacts, and engagement in social activities (4, 5, 11). However, there is little consensus on these assessment methods. In this study, we used self-report questionnaires to assess social behavior, such as the frequency of outings, contact, and cognitive or social activity. The increasing number of older adults who live alone and are housebound has led to a higher proportion of them experiencing social isolation. Therefore, quantifying social isolation is essential better to understand the relationship between social isolation and health. Additionally, developing simple methods to quantify the degree of social activity is an important yet challenging task for researchers. Since wearable sensors can remotely collect data on daily life and behavior in real time, they may be used for early detection of social issues, including social isolation (21, 22). Several studies have assessed loneliness or social isolation by analyzing data on household behavior, physical activity, or sleep from smartphones or wearable sensors (23–28). These results show that eating behavior, mobility, and sleep quality are associated with social isolation (23, 24). On the other hand, the association of objectively measured physical activity with social isolation showed inconsistent findings (25–27). Moreover, making and receiving phone calls, social engagement on the web, computer usage, social interaction, smartphone usage, physical activity, or sleep duration are associated with loneliness (28). Therefore, objectively measuring various lifestyles using sensing techniques may be a surrogate parameter for social isolation. Conversation is an essential lifestyle factor for older adults to develop social relationships and preserve brain function, mental health, and quality of life (29, 30). Only three studies have measured conversation time using smartphone data collected in the ambient environment as a conversation and examined the association between conversation time and loneliness (18–20). Two studies found no significant association between conversation time and loneliness in young adults or patients with schizophrenia spectrum disorders (18, 19). In contrast, the remaining research showed a substantial association of conversation time with depression in students (20). Our results showed a significant association of objective conversation time with social behavior. We suggest that objectively measured conversation time using a wearable sensor could be a simple and non-invasive method to assess the social behavior of older adults living in the community. Social isolation is associated with an increased risk of poor health and leads to increased personal and societal costs by increasing the demands for healthcare resources, including the management of acute, chronic, and consumptive illnesses, the use of emergency medical care, and institutionalization (31–33). Therefore, early detection and management of social isolation are crucial for the prevention of various diseases, including dementia, and decreased need for hospital visits, hospitalization, and institutionalization. Social implementation of conversation time measuring technology has the potential to facilitate screening for social isolation among community-dwelling older adults. Additionally, this tool may reduce the demands for human resources, resulting in decreased time or cost burden on individuals, medical staff, or researchers in research or clinical settings.

Several limitations should be noted in this study. First, since the aim of this study is to clarify the association of lifestyle factors with cognitive function, established self-report questionnaires of social isolation, such as the Lubben Social Network Scale (34) and Social Disconnectedness Scale (35), were not included in this study design. Second, we did not consider the effect of family structure on conversation time. Nonetheless, there was no significant association between conversation time and superficial contact with friends or relatives. Third, the accuracy of conversation time detection was verified by comparing the wristband sensor data with video observational data, but television viewing or radio-listening sounds could have been detected as conversation. Fourth, it is important to collect only conversations with good reciprocity and alternation between social actors. However, our sensor technology cannot distinguish between one-way and reciprocal conversations. In this study, we considered that participation in both one-way or reciprocal conversation was necessary for social activity. Further technical developments are required to identify only reciprocal conversations. Fifth, the number of valid data form self-report questionnaire was different for each item due to missing data.

## Conclusion

To our knowledge, this is the first study to report the association between objective conversation time and the frequency of engagement in community activities and lessons or classes. Objectively measured conversation time using a wearable sensor could be a valuable parameter for determining social isolation in large cohort studies.

## Data Availability

The raw data used in this study contains sensitive and identifying information on individuals, including sex, age, and education level, that could compromise the privacy of research participants. However, the data that support the findings of this study are available upon ethical approval by the local ethics committee of the Oita University Hospital. Please contact the corresponding author. Email: rinrikenkyu@oita-u.ac.jp.
